# Quantitative and Targeted Regulation of Ferroptosis in Bladder Cancer: Preclinical Study

**DOI:** 10.1111/jcmm.71333

**Published:** 2026-08-26

**Authors:** Ying Dong, Chaojie Xu, Bing Yan, Shuanzhu Mou, Chen Li, Yuchen Liu

**Affiliations:** ^1^ Shenzhen University Medical School, The First Affiliated Hospital of Shenzhen University, Shenzhen Second People's Hospital Shenzhen University Shenzhen China; ^2^ Shenzhen Institute of Translational Medicine, Urology of Shenzhen Second People's Hospital, The First Affiliated Hospital of Shenzhen University, Health Science Center Shenzhen University Shenzhen China; ^3^ Shenzhen Institute of Synthetic Biology, Shenzhen Institutes of Advanced Technology Chinese Academy of Sciences Shenzhen China; ^4^ Department of Urology, Peking University First Hospital Peking University Beijing China; ^5^ Department of Pharmacy The First Affiliated Hospital of Guangxi Medical University Nanning China

**Keywords:** BECN1, bladder cancer, CRISPR‐dCas9, ferroptosis, hTERT, OTUB1

## Abstract

The activation of ferroptosis, a cell death mechanism driven by excessive ferrous ions (Fe^2+^) and lipid peroxides, has emerged as a promising target for cancer treatment. However, in the case of quantitative regulation of target genes, it remains uncertain whether ferroptosis can be induced in bladder cancer (BCa) cells without affecting normal ones. We investigated this using an innovative CRISPR‐dCas9 system to upregulate and downregulate the ferroptosis‐related gene BECN1 and OTUB1, respectively. We identified two genes that can affect and promote ferroptosis‐related pathways, analysing their expression in bladder tissue through The Cancer Genome Atlas. Our unique CRISPR‐dCas9 technology, under the control of an hTERT promoter, selectively adjusted BECN1 and OTUB1 expression exclusively in cancer cells. RT‐qPCR and western blotting demonstrated significant alterations in the expression of GPX4 and SLC7A11, proteins strongly associated with ferroptosis, in BCa cells, while normal bladder cells remained unaffected. We developed a quantitative model based on synthetic biology principles to describe the regulatory relationships between the ferroptosis‐related genes BECN1 and OTUB1 and their downstream targets GPX4 and SLC7A11 in bladder cancer cells. The model establishes a direct proportional relationship between BECN1 upregulation and decreased GPX4 expression, and between OTUB1 downregulation and decreased SLC7A11 expression. In vitro experiments revealed reduced viability, proliferation, migration, and invasion in UMUC‐3 and T24 BCa cells. Importantly, Fer‐1 and DFO rescued the viability loss, and C11‐BODIPY staining confirmed increased lipid ROS accumulation, supporting ferroptosis‐associated cell death following BECN1/OTUB1 regulation. In vivo xenograft experiments showed that BECN1 upregulation or OTUB1 downregulation suppressed tumour growth. Tumour‐tissue immunofluorescence further showed reduced GPX4 expression in BECN1‐upregulated tumours and reduced SLC7A11 expression in OTUB1‐downregulated tumours, supporting suppression of the GPX4/SLC7A11 ferroptosis‐protective axis in vivo. The quantitative equation derived from our data suggests that the induction of ferroptosis in bladder cancer cells can be effectively modulated by these two genes, and the experimental results also indicate our system can modulate these two genes to affect the function of BCa cells without affecting the normal cells, offering a promising new direction for the development of targeted therapy for bladder cancer.

**Abbreviations**
ATCC: American Type Culture Collection
ATG: anti‐thymocyte glubulin
BCa: bladder cancer
CCK‐8: cell counting kit‐8
cDNA: complementary deoxyribonucleic acid
CRISPR‐dCas9: clustered regularly interspaced short palindromic repeats‐dead associated protein 9
DAPI: 4′,6‐diamidino‐2‐phenylindole
EdU: 5‐ethynyl‐2′‐deoxyuridine
FBS: fetal bovine serum
GAPDH: glyceraldehyde‐3‐phosphate dehydrogenase
HRP: horseradish peroxidase
hTERT: human‐telomerase reverse transcriptase
MEM: minimum essential medium
NC: negative control
PBS: phosphate buffered saline
RNA: ribonucleic acid
RT‐qPCR: real‐time quantitative polymerase chain reaction
sgRNA: small guide ribonucleic acid
TCGA: the cancer genome atlas
VP64: virus protein64
WB: western blotting

## 1 Introduction

Bladder cancer (BCa) accounts for 4.0% of the total global cancer incidence rate, which makes it the tenth most common cancer as of 2023 [1]. In China, BCa is the most common urological tumour type and has the highest mortality rate of urological cancers. Despite this, there remains a lack of effective treatments for mid‐ to late‐stage BCa, resulting in poor prognosis and high mortality. Chemotherapy represents the primary treatment route for bladder tumours; however, because of the resistance of cancer cells to chemotherapeutics, this treatment often fails to achieve good results in patients with advanced tumours [2]. Therefore, exploring the pathogenesis of BCa to identify new treatment methods is of great importance, as is the exploration of new technological approaches to aid the precise treatment of BCa.

BECN1 is one of the numbers of an evolutionarily conserved family of proteins that regulates autophagy‐dependent or autophagy‐independent cellular metabolism [3, 4]. BECN1, a core component of the BECN1‐PIK3R3‐PIK3R4 complex which is indispensable for autophagy induction, consists of three structural domains: BH3, the coiled‐coil structural domain (CCD), and BARA (which includes ECD). The binding of other proteins to these structural domains is essential for complex formation, and thus underlies the role of BECN1 in autophagy initiation. For example, phosphatidylinositol 3 kinase III (PtdIns3P) interacts with CCD and ECD and facilitates the recruitment of ATG proteins to phagocytic clusters [3, 5]. In cancer, BECN1's role is particularly significant. It can function as a tumour suppressor, where its autophagic activity helps to limit cell proliferation by maintaining cellular integrity and preventing genomic instability. Reduced expression of BECN1 has been associated with various cancers, suggesting that its autophagy‐regulating function is crucial in suppressing tumour growth [6, 7]. Conversely, in some cancer types, autophagy can support tumour cell survival, indicating a complex relationship between BECN1, autophagy, and cell proliferation. BECN1 is also capable of driving ferroptosis, making it an interesting gene from the perspective of cancer treatment, especially since BCa upregulation of BECN1 may affect the expression of other proteins involved in ferroptosis [6]. It has been demonstrated that upregulation of BECN1 leads to reduced expression of glutathione peroxidase, GPX4, which results in reduced scavenging of lipid peroxides by the cell membrane and leads to excessive accumulation of oxidized substances on the cell membrane [8, 9]. In addition, regulation of BECN1 can promote the occurrence of ferroptosis in cells to treat breast cancer [10]. However, the role of BECN1 in BCa cells remains unclear.

OTUB1 is an ovarian tumour (OTU) family member deubiquitinating enzyme that promotes tumour cell survival and proliferation by regulating the activities of Mdm2 and Mdmx [11]. OTUB1 is thought to regulate many cancer‐related signalling pathways, including MAPK, ERa, epithelial‐mesenchymal transition (EMT), RHOa, mTORC1, FOXM1, and P53, as well as invasiveness and drug resistance [12]. OTUB1 is overexpressed in a variety of human cancers, and elevated OTUB1 expression is associated with tumour grade, invasiveness, and metastasis [13]. Some studies have found that depletion of endogenous OTUB1 decreases SLC7A11 in human cancer cells, and that OTUB1 may directly interact with SLC7A11 and affect the stability of SLC7A11 [14]. The cystine/glutamate anti‐transporter protein SLC7A11/xCT functions to import cystine into the cell for glutathione biosynthesis, and this transporter protein has been shown to be in an overexpressed state in cancer [15]. If SLC7A11 is destabilized, it can ultimately lead to loss of intracellular cystine levels and depletion of intracellular glutathione biosynthesis, promoting iron death [16]. Therefore, by targeting and down‐regulating the OTUB1 gene, it can promote lipid peroxidation accumulation and death in bladder cancer cells by affecting the expression of SLC7A11.

Human telomerase reverse transcriptase (hTERT) is highly expressed and active in cancer cells [17], while being mostly silenced in normal cells [18]. Experimental evidence demonstrates that an artificial hTERT promoter can be used to efficiently drive high levels of expression of the downstream gene in cancer cells [19]. By utilizing this artificial hTERT promoter and exploiting the higher activity of telomerase in cancerous cells, we can specifically drive gene expression in cancer cells while minimally altering expression in normal cells. CRISPR‐dCas9, a modified CRISPR‐Cas9 system containing an inactive, or ‘dead’ Cas9 component, is a novel gene‐targeting technique that can modulate the expression of a specific target gene [20, 21].

This study utilized CRISPR‐dCas9 under the control of the hTERT promoter to drive cancer cell‐specific upregulation of BECN1 and downregulation of OTUB1. The aim was to explore whether targeted regulation of BECN1 and OTUB1 could induce lipid peroxidation and ferroptosis in bladder cancer cells without affecting normal BCa cells.

## 2 Methods and Materials

### 2.1 Acquisition and Culture of Cells

The human BCa uroepithelial cells T24 and UMUC‐3, and the human normal uroepithelial cells SV‐HUC1, all obtained from ATCC, were used in this experiment. Cells were cultured at 37°C with 5% CO_2_. McCoy's 5A medium was used for T24, MEM was used for UMUC‐3, and F‐12k was used for SV‐HUC‐1. The complete medium comprised 90% medium, 10% FBS, and 1% double antibody.

### 2.2 Gene Screening and Construction of Viral Backbone

Human genes associated with ferroptosis were identified using FerrDb (https://www.zhounan.org/ferrdb/legacy/index.html) [22]. The driver target gene BECN1 and its downstream gene GPX4 were screened to determine the reliability of these genes and their related downstream pathways. Subsequently, the feasibility of down‐regulated genes and their associated downstream genes was assessed through references, leading to the identification of the target gene OTUB1 and its downstream gene SLC7A11 [23]. To test the potential specificity of the selected gene targets, we used The Cancer Genome Atlas (TCGA) database to examine the expression levels of BECN1 and OTUB1 [24], finding significant differences in their expression in BCa tissues compared to normal tissues. Vectors consisting of hTERT‐promoter‐dCas9‐VP64 and hTERT‐promoter‐dCas9‐KRAB with puromycin resistance, along with different sgRNAs with neomycin resistance, were designed to target BECN1 and OTUB1. The sequence of hTERT‐promoter‐dCas9‐VP64‐puro and hTERT‐promoter‐dCas9‐KRAB‐puro are provided in Table S1. The sequences of the sgRNAs targeting BECN1 and OTUB1 are given in Table S2. All the above constructs were designed by Shanghai Genechem Co. Ltd.

### 2.3 Virus Transfection, Regulation of Related Genes

The cultured T24, UMUC‐3, and SV‐HUC1 cells were transfected with hTERT‐promoter‐dCas9‐VP64 and hTERT‐promoter‐dCas9‐KRAB adenovirus respectively. The virus vector map is in Table S3. After 72 h the cells were screened using 1 μg/mL puromycin to select for cells transfected with the virus. Five days later, the cells were again transfected with adenovirus carrying sgRNA (U6‐sgRNA‐SV40‐Neomycin). After 72 h the cells were again screened for transfection by adding 1 μg/mL puromycin and 1 mg/mL neomycin to the complete medium. The resulting cells were those which stably expressed the transfected constructs.

### 2.4 RNA Extraction and RT‐qPCR

RNA was extracted from cells using RNA kits (Vazyme, Nanjing). The extracted RNA was then reverse transcribed into cDNA using reverse transcription kits (YEASEN, Shanghai). Samples were spiked separately according to the manufacturer's instructions, and the expression of both housekeeping and target genes was measured using PCR. Five replicates were conducted for every sample. The 2^−ΔΔ*Ct*^ method was used to quantify the relative expression levels of target genes in cells. Primers for the internal reference gene and target gene were designed using PrimerBank (PrimerBank ED, Harvard). The following sequences were used: GAPDH, F: CCTTCCGTGTTCCTACCC, R: CAACCTGGTCCTCAGTGTAG; BECN1, F: AACATATCCTCATCTCAGTAA, R: TCCTTTCCCTTTCTTCTC; GPX4, F: GAGGCAAGACCGAAGTAAACTAC, R: CCGAACTGGTTACACGGGAA; OTUB1, F: TTCTGTGGTTGTAAATGG R: CAGGAGTAGATAGGTGAA; SLC7A11: F: TCTCCAAAGGAGGTTACCTGC R: AGACTCCCCTCAGTAAAGTGAC.

### 2.5 Western Blotting to Verify the Expression of Related Proteins

Proteins were extracted from the cells, and the protein concentration was measured using aBCA box (Beyoncé Protein Kit, Guangdong). The corresponding amount of protein was then added to the precast gel and transferred onto a polyvinylidene fluoride membrane for transfer blotting. Antibodies for the target genes, BECN1, GPX4, OTUB1 and SLC7A11, and an antibody against the internal reference actin, were used to quantify the levels of the corresponding proteins. Rabbit antibodies were used as the secondary antibodies. The antibodies were first diluted using Universal Protein Diluent at the following concentrations: anti‐BECN1 (Abclone, China), 1:2000; anti‐GPX4 (Abcam, American), 1:1000; anti‐OTUB1 (Abclone, China), 1:3000; anti‐SLC7A11 (Abcam, American), 1:500; anti‐β‐actin (Abcam. American), 1:7000; HRP‐linked anti‐IgG (Abcam, American), 1:8000. The antibodies were then added to the membranes, which were incubated overnight at 4°C. The following day the membranes were incubated with secondary antibodies at room temperature for 1 h. Immunoreactive bands were observed using the Beyoncé Ultrasensitive Developer, and the area of the immunobands was analysed using the Image J software.

### 2.6 Quantitative Modelling of Ferroptosis Regulation

To quantify the impact of BECN1 and OTUB1 regulation on ferroptosis in bladder cancer cells, we developed a mathematical model reflecting the relationship between these genes and their downstream targets GPX4 and SLC7A11. The changes in GPX4 and SLC7A11 expression levels were modelled using Hill equations as follows:

$$d\left[GPX4\right]/\mathrm{dt}=-V_{\text{max1}}\cdot {\left[BECN1\right]}^{\text{n1}}/\left(K_{\text{d1}}+{\left[BECN1\right]}^{\text{n1}}\right)$$

$$d\left[SLC711A\right]/\mathrm{dt}=V_{\text{max2}}\cdot {\left[OTUB1\right]}^{\text{n2}}/\left(K_{\text{d2}}+{\left[OTUB1\right]}^{\text{n2}}\right)$$

where V_max1_, K_d1_, and n_1_ are constants derived from fitting the GPX4 model to experimental data for BECN1, and V_max2_, K_d2_, and n_2_ are constants derived from fitting the SLC7A11 model to experimental data for OTUB1. This quantitative approach allows us to predict the extent of ferroptosis induction based on the modulation of these genes.

### 2.7 Annexin V/PI Cell‐Death Assay

Annexin V/PI staining was used to assess cell‐death changes after BECN1 upregulation or OTUB1 downregulation. Cells stably expressing the CRISPR constructs were collected and washed using PBS at 4°C, before being by resuspended by adding pre‐chilled 1× Annexin V Binding Buffer to the cell precipitates. Then, 5 μL Annexin V + 5 μL PI, 5 μL Annexin V, and 5 μL PI were added to the tubes respectively, and the cells were incubated on ice less than 1 h in the dark. Cells survival rate was then measured using Cell flow instrumentation.

### 2.8 CCK8 Assay

Cells were inoculated into 96‐well plates at a density of 8 × 10^3^ per well and cultured overnight in complete medium. After the cells were adhered, we chose this time as the 0 h, then select 0, 24, and 48 h pre‐prepared CCK8 reagent (CCK‐8: serum‐free medium = 1:10) was added to the wells under light‐proof conditions. The plates were incubated for 2 h under light‐proof conditions, after which the enzyme marker was added and the absorbance value (OD) of each well was measured at 450 nm.

### 2.9 Ferroptosis Inhibitor Rescue Assay

To determine whether the reduced viability induced by BECN1 upregulation or OTUB1 downregulation was associated with ferroptosis, rescue experiments were performed using the ferroptosis inhibitor Ferrostatin‐1 (Fer‐1) and the iron chelator deferoxamine (DFO). Stable T24 and UMUC‐3 cells expressing the indicated CRISPR‐dCas9 constructs were seeded into 96‐well plates and treated with Fer‐1 (5 μM) or DFO (200 μM) for 24 h. Cell viability was then measured using the CCK‐8 assay. Absorbance at 450 nm was recorded using a microplate reader. Relative cell viability was normalized to the corresponding NC group.

### 2.10 C11‐BODIPY Lipid ROS Assay

Lipid peroxidation was assessed using C11‐BODIPY 581/591 staining. Briefly, T24 cells from the indicated groups were incubated with C11‐BODIPY 581/591 (2 μM) at 37°C for 20 min in the dark. After staining, cells were washed with PBS and analysed by fluorescence microscopy and flow cytometry. Oxidation of C11‐BODIPY was evaluated by the shift from red to green fluorescence, and lipid ROS levels were quantified as the relative green fluorescence intensity or the percentage of oxidized C11‐BODIPY‐positive cells. For rescue experiments, cells were pretreated with Fer‐1 or DFO before C11‐BODIPY staining for 24 h.

### 2.11 EDU Assay

Cells were inoculated into 6‐well plates at a density of 3 × 105 per well, cultured using complete medium. After the cells were adhered, they were treated using the EdUCell Kit (Beyoncé, Shanghai), following the manufacturer's instructions. The nuclei were stained by the addition of fluorescent dyes or DAPI, and the staining signal of EdU was detected using a fluorescence microscope. The plates were photographed and recorded.

### 2.12 Wound Healing Assay

Cells stably expressing the CRISPR constructs were inoculated into 12‐well cell plates. After the cells were flattened in the well plates, the flat cell layer was scratched using a 200 μL pipette wall tip and then we used the PBS to wash off the cell debris. Afterwards, the cells continued to be cultured using serum‐free medium. The area in which the cells were scratched was photographed and recorded at 0, 24, and 48 h after the scratch was made.

### 2.13 Transwell Assay

Cells stably expressing the CRISPR constructs were resuspended in serum‐free medium to obtain a concentration of 1 × 10^5^/mL cells suspension. A sample of 200 μL was added to the upper chamber of the transwell that was coated with matrix gel. Complete medium containing 20% FBS was added to the lower chamber. After 24 h of incubation, the chambers can be removed, washed, fixed, and stained (0.1% Crystalline Violet). Finally, the cells were observed under a microscope and photographed, and the number of cells in each chamber was calculated separately using ImageJ software.

### 2.14 In Vivo Experiment in Mice

The use of mice in this study was approved by the Animal Ethics Committee. Experiments were performed using transfected T24 cells stably expressing the CRISPR construct. 24 BALB/c nude mice (4–5 weeks) were divided into four groups: VP64‐NC, VP64‐BECN1, KRAB‐NC, KRAB‐OTUB1. The T24 cells were resuspended in an equal volume of cold PBS and mixed with stromal gel. Mice were injected with 100 w units of cell suspension in the right axilla, and tumour volume was measured and calculated at 4‐day intervals using the following formula: tumour volume = length × width × 0.5 × width. Mice were executed 1 month after injection, and tumours were preserved.

### 2.15 Immunofluorescence Staining of Paraffin‐Embedded Tumour Tissues

Xenograft tumour tissues were collected from mice, fixed in 4% paraformaldehyde, embedded in paraffin, and sectioned at 4 μm. The paraffin sections were deparaffinized in xylene, rehydrated through a graded ethanol series, and subjected to antigen retrieval using citrate antigen retrieval buffer. After cooling to room temperature, the sections were washed with PBS, permeabilized with 0.3% Triton X‐100, and blocked with 5% bovine serum albumin at room temperature. The sections were then incubated with primary antibodies against GPX4 and SLC7A11 at 4°C for 16 h. After washing with PBS, the sections were incubated with the corresponding fluorescent secondary antibodies at room temperature in the dark. Nuclei were counterstained with DAPI. Images were captured using a fluorescence microscope under identical acquisition settings for each marker. The fluorescence intensity of GPX4 and SLC7A11 was quantified using ImageJ software in randomly selected non‐necrotic tumour fields and normalized to the corresponding NC group.

### 2.16 Statistical Analysis

All quantitative data are presented as mean ± SD from at least three independent experiments unless otherwise indicated. Statistical analyses were performed using GraphPad Prism software. Comparisons between two groups were performed using two‐tailed Student's *t*‐test, and comparisons among multiple groups were performed using one‐way ANOVA followed by appropriate multiple‐comparison tests. *p* < 0.05 was considered statistically significant. ns, not significant; *p < 0.05; **p < 0.01; ***p < 0.001; ****p < 0.0001.

## 3 Results

### 3.1 The Expression of BECN1 and OTUB1 Protein Was Significantly Altered in Cancer Tissues

**FIGURE 1:**
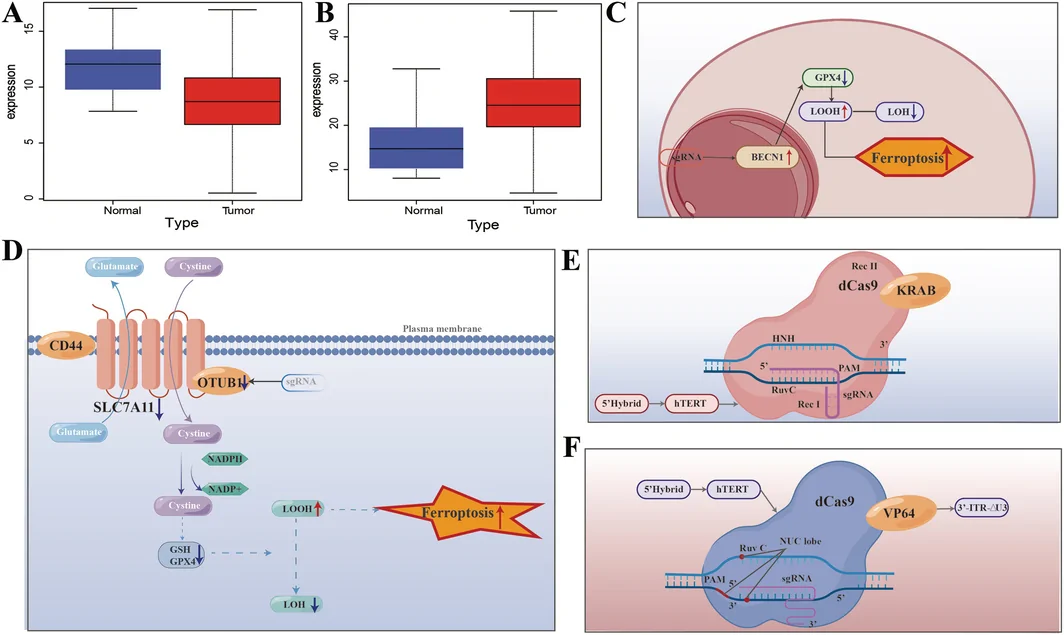
(A, B) Expression of BECN1 and OTUB1 in BCa tissues compared to normal tissue. (C) BECN1 modulates ferroptosis‐associated lipid peroxidation. (D) SLC7A11‐centered antioxidant axis regulates ferroptotic susceptibility (E, F) Mechanism of the hTERT‐CRISPR‐dCas9‐VP64 and hTERT‐CRISPR‐dCas9‐KRAB.

To investigate the potential effect of the BECN1 and OTUB1 genes on BCa cells, we downloaded data from the TCGA database and examined BECN1 and OTUB1 expression levels. We found that, compared to normal tissues, BECN1 expression levels were significantly lower in BCa tissues and there was a statistically significant decrease in OTUB1 expression levels (*p* < 0.05) (Figure 1A,B). This result indicates that BECN1 and OTUB1 may play important roles in BCa. We determined the likely pathways through which BECN1 and OTUB1 affect ferroptosis by reviewing the relevant literature; the resulting pathway is shown in Figure 1C,D.

### 3.2 Roles of BECN1 and OTUB1 in Cancerous Cells

**FIGURE 2:**
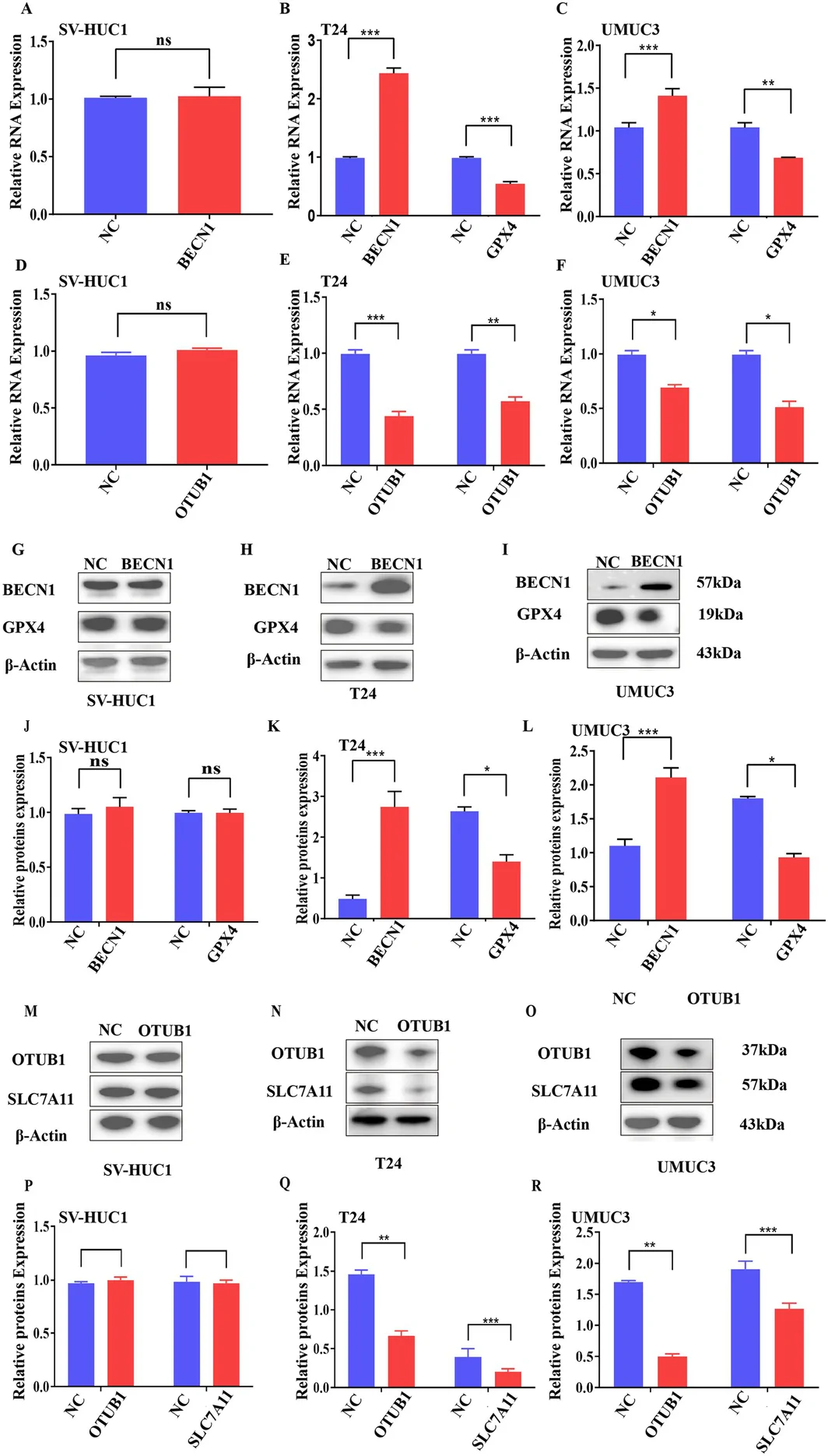
(A, D) RT‐qPCR results showing the relative expression of BECN1 and OTUB1 in SV‐HUC1 cells. (B, C, E, F) RT‐qPCR results showing the relative expression of BECN1, GPX4, OTUB1 and SLC7A11 in T24 and UMUC3 cells. NC; negative control. (G–R) Western blotting results, including both blotting images and relative protein expression, for the expression of BECN1, GPX4, OTUB1 and SLC7A11 in T24 (H, K, N, Q), UMUC3 (I, L, O, R), and SV‐HUC1 (G, J, M, P) cells. ns, no significant; *p < 0.05, **p < 0.01 ***p < 0.001.

**FIGURE 3:**
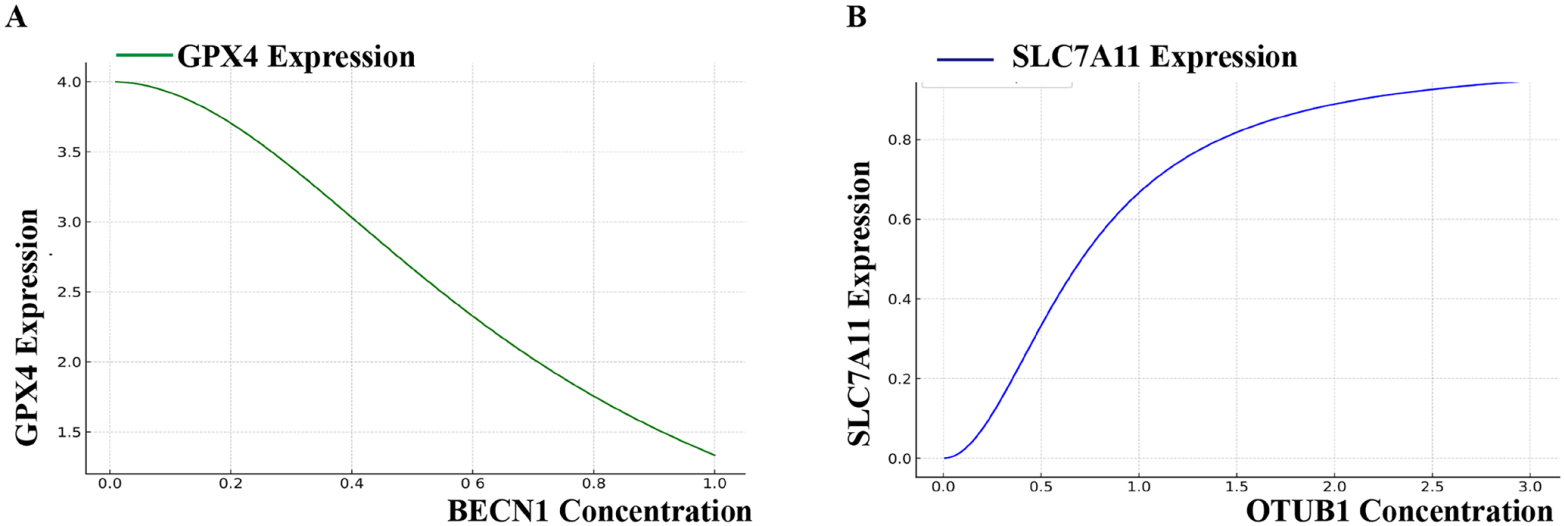
(A) GPX4 expression as a Function of BECN1 concentration. (B) SLC7A11 expression as a Function of OTUB1 concentration.

The CRISPR‐dCas9 system (Figure 1E,F) was used to specifically regulate BECN1 and OTUB1 in cancer cells. The sgRNA targeted the dCas9 to BENC1, where VP64 promoted upregulation, and to OTUB1, where KRAB promoted downregulation. The hTERT promoter led to higher expression of the construct in cancer cells compared to normal cells. Cas9 is a nucleic acid endonuclease that, when its two catalytic sites (RuvC and HNH) are mutated, becomes an inactivated form known as dCas9. This form loses its DNA‐cleaving ability but retains the capacity to bind specifically to target DNA sequences under the guidance of a guide RNA (gRNA). KRAB is a potent transcriptional repressor domain found in various zinc‐finger transcription factors, capable of recruiting transcriptional repression complexes that compact the chromatin structure, thereby suppressing gene expression. In contrast, VP64 is a transcriptional activator that effectively recruits transcriptional cofactors and, through synergistic interactions with other activators, facilitates RNA polymerase II binding, thereby upregulating target gene expression. Leveraging the high expression of hTERT in tumour cells, combining the hTERT promoter with a tool capable of specific gene upregulation and downregulation allows for selective, high‐efficiency expression within tumour cells, enabling targeted regulation of downstream genes and achieving precise therapeutic effects in tumour cells. The constructed viral tools were transfected into T24, UMUC‐3, and SV‐HUC1 cells. After screening for stable transgenic strains, the infection efficiency of the virus was verified at both the RNA and protein levels using RT‐qPCR and western blotting, respectively. The expression of BECN1 and OTUB1, our desired target genes, was not affected by transfection with the virus in the normal bladder cell line, SV‐HUC1 (Figure 2A,D,F,G,J,M). Under consistent viral transfection conditions, BECN1 was upregulated and OTUB1 was downregulated at both the RNA and protein levels in cancer cell lines T24 and UMUC‐3. Intriguingly, the expression of the BECN1 downstream protein GPX4 and OTUB1 downstream protein SLC7A11, both highly associated with ferroptosis, were also relatively reduced (Figure 2B,C,E,F,H,I,K,L,N,O,Q,R). Our quantitative model accurately reflected the observed experimental data. The fitting process revealed that the constants K_d1_ and K_d2_ are both significant, indicating that the modulation of BECN1 and OTUB1 has a strong and quantifiable impact on the expression of GPX4 and SLC7A11, respectively (Figure 3A,B). The model predicts that upregulation of BECN1 and downregulation of OTUB1 contribute to the accumulation of lipid peroxides and depletion of glutathione, effectively promoting ferroptosis in bladder cancer cells.

### 3.3 Regulation of BECN1 and OTUB1 Affects the Proliferation of Cancerous Cells

**FIGURE 4:**
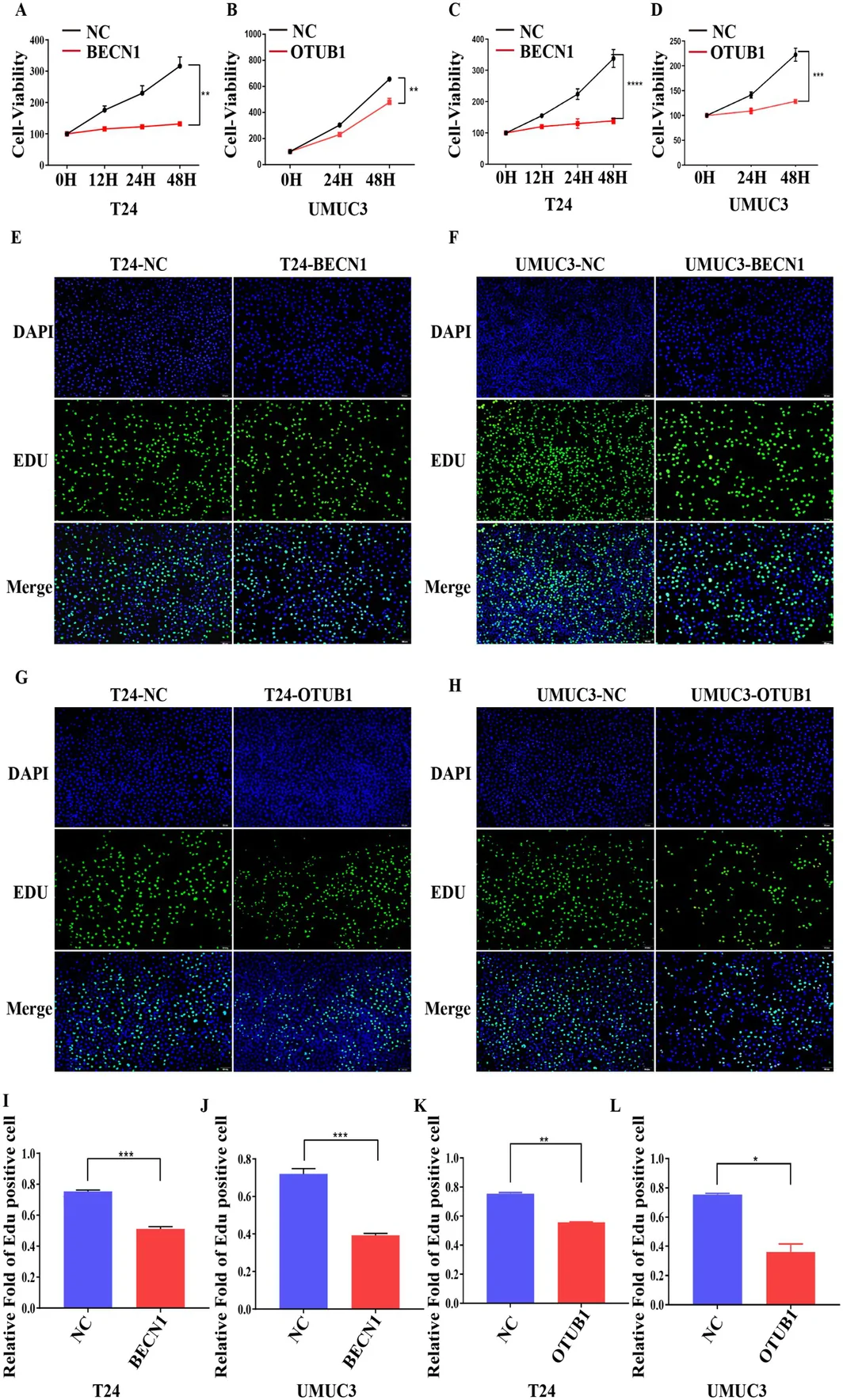
(A–D) CCK‐8 assay results showing the proliferative capacity of stable transgenic strains compared to cells of NC. (E–L) EdU assay results showing the proliferative capacity of stable transgenic cells (T24: E, G, I, K; UMUC3: F, H, J, L) compared to normal cells. ns, no significant; *p < 0.05, **p < 0.01, ***p < 0.001, ****p < 0.0001.

The CCK‐8 assay was used to quantify cell proliferation and viability, chosen for its sensitivity, reliability, and simplicity. The CCK‐8 assay showed that BCa cells with high expression of BECN1 and low expression of OTUB1 exhibited significantly less proliferation at 48 h compared to un‐transfected cells, indicating inhibited cell proliferation (Figure 4A–D). Similarly, the results of EdU experiments demonstrated that the proliferation of T24 and UMUC‐3 cells in the transfected group was significantly inhibited compared to the control group (Figure 4E–L).

### 3.4 Ferroptosis Inhibitor Rescue Confirms Ferroptosis‐Associated Lipid Peroxidation

**FIGURE 5:**
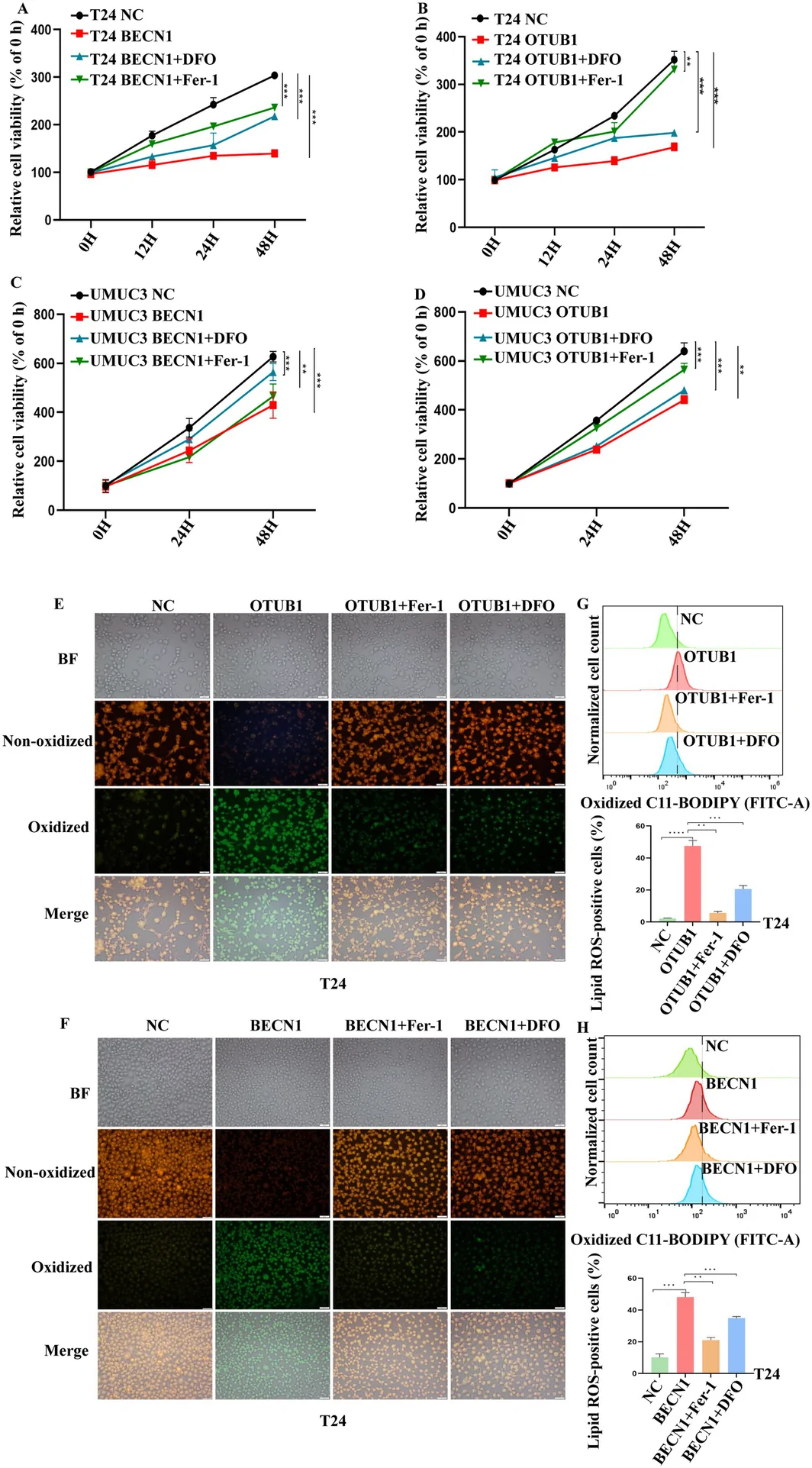
Ferroptosis inhibitor rescue and lipid ROS validation in bladder cancer cells. (A–D) CCK‐8 assays showing the effects of Ferrostatin‐1 (Fer‐1) and deferoxamine (DFO) on cell viability in T24 and UMUC‐3 cells after BECN1 upregulation or OTUB1 downregulation. Fer‐1 and DFO partially rescued the reduction in cell viability induced by BECN1 or OTUB1 regulation. (E, F) Representative C11‐BODIPY 581/591 fluorescence images showing lipid ROS accumulation in T24 cells. BECN1 upregulation or OTUB1 downregulation increased the oxidized C11‐BODIPY signal, whereas Fer‐1 and DFO treatment reduced lipid ROS accumulation. (G, H) Flow cytometric analysis and quantification of oxidized C11‐BODIPY‐positive T24 cells. Lipid ROS‐positive cells were quantified as the percentage of oxidized C11‐BODIPY‐positive cells. Data are presented as mean ± SD. ns, not significant; **p < 0.01; ***p < 0.001; ****p < 0.001.

To further determine whether the reduced viability caused by BECN1 upregulation or OTUB1 downregulation was attributable to ferroptosis rather than a nonspecific growth‐inhibitory effect, we performed ferroptosis inhibitor rescue experiments. In both T24 and UMUC‐3 cells, regulation of BECN1 or OTUB1 markedly reduced cell viability compared with the corresponding NC group. Notably, treatment with Ferrostatin‐1 partially restored cell viability, and DFO also attenuated the viability loss induced by BECN1/OTUB1 regulation, although the magnitude of rescue was more modest in some conditions (Figure 5A–D). These results indicate that the growth‐suppressive effect of BECN1/OTUB1 regulation is at least partly dependent on ferroptosis‐associated lipid peroxidation and iron availability. We next examined lipid ROS accumulation using C11‐BODIPY 581/591 staining. In T24 cells, BECN1 upregulation or OTUB1 downregulation increased the oxidized C11‐BODIPY signal, indicating enhanced lipid peroxidation. Consistently, Fer‐1 and DFO treatment reduced the oxidized C11‐BODIPY signal, as shown by both fluorescence microscopy and flow cytometry (Figure 5E–H). Together, these findings provide direct evidence that BECN1/OTUB1 regulation promotes lipid peroxidation and ferroptosis‐associated cell death in bladder cancer cells.

### 3.5 Regulation of BECN1 and OTUB1 Affects the Migration and Invasion of Cancer Cells

**FIGURE 6:**
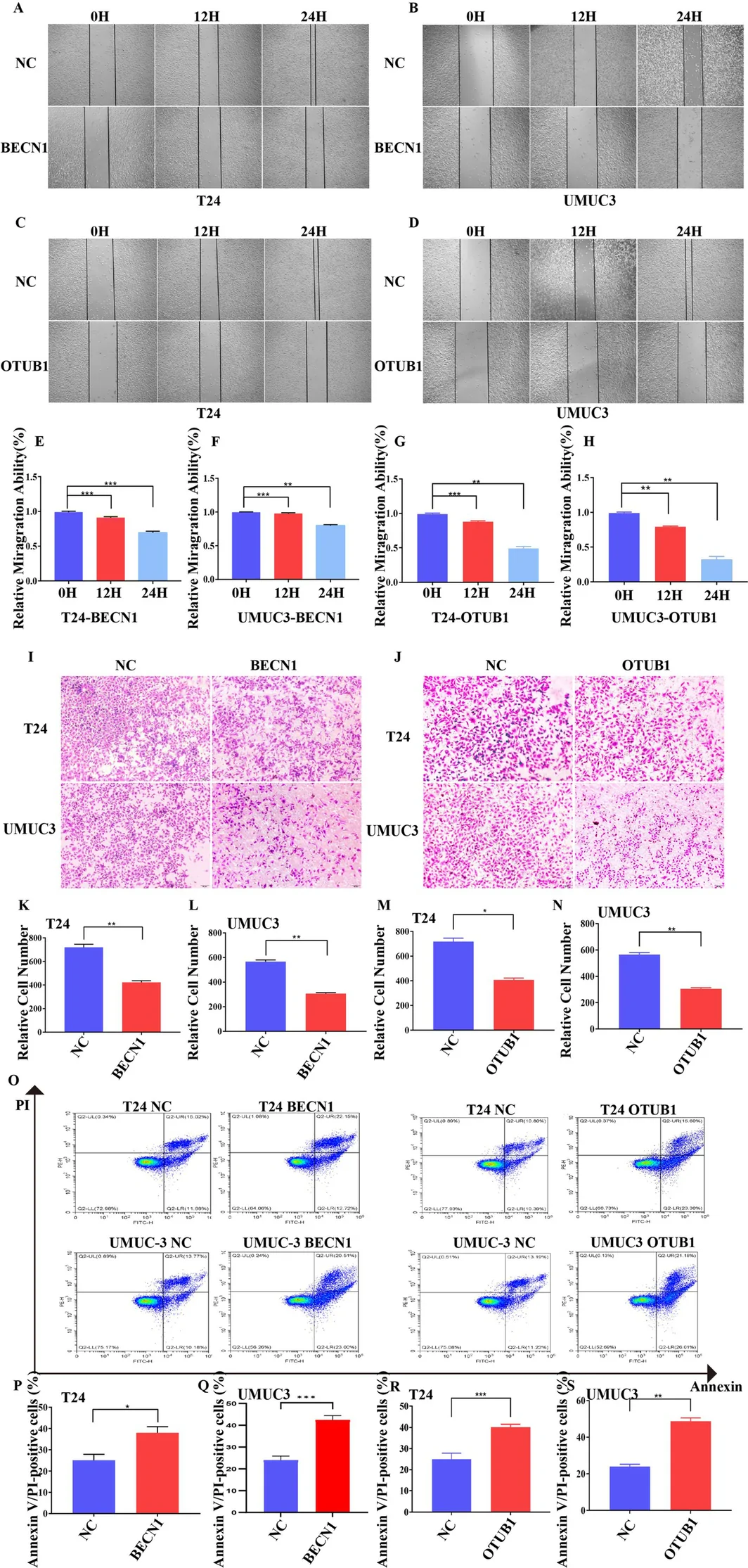
(A–D) Wound healing assay results show the migratory capacity of stable infected T24 (A, B, E, F) and UMUC3 (C, D, G, H) cells. (I–N) Transwell assay results show the invasive capacity of stable infected T24 and UMUC3 cells. (O–S) Annexin V/PI cell‐death assay. (O) Representative flow cytometry plots. (P–S) Quantification of Annexin V/PI‐positive cells. NC: ns *p < 0.05, **p < 0.01 ***p < 0.001.

We further investigated whether cancerous cells with upregulated BECN1 and downregulated OTUB1 showed changes in migration and invasion compared to untreated BCa cells. A wound healing assay, which mimics a wound in a cell monolayer and observes how cells migrate to close this gap, was used to assess migratory ability. The assay showed that the migratory ability of cells in the transfected group was significantly inhibited compared to the NC group (Figure 6A–H). A transwell assay was used to verify the invasive ability of the cells, revealing that the invasive ability of the cells in the experimental group was inhibited compared to the control group (Figure 6I–N).

### 3.6 Regulation of BECN1 and OTUB1 Increases Annexin V/PI‐Positive Cell Death in Bladder Cancer Cells

Annexin V/PI staining was used to evaluate cell‐death changes after BECN1 upregulation or OTUB1 downregulation. As shown in (Figure 6O–S), the proportion of Annexin V/PI‐positive T24 and UMUC‐3 cells was significantly increased compared with the corresponding NC groups. Because Annexin V/PI staining is not specific for apoptosis and can reflect multiple forms of cell death, these data were interpreted as Annexin V/PI‐positive cell death rather than definitive apoptotic death. Together with the ferroptosis inhibitor rescue and lipid ROS assays, these findings suggest that BECN1/OTUB1 regulation induces ferroptosis‐associated cell death in bladder cancer cells. As a supporting analysis, Fer‐1 treatment reduced Annexin V‐positive cell death in BECN1‐upregulated T24 cells (Figure S1).

### 3.7 BECN1 and OTUB1 Regulation Suppresses Xenograft Tumour Growth and Inhibits the GPX4/SLC7A11 Ferroptosis‐Protective Axis In Vivo

**FIGURE 7:**
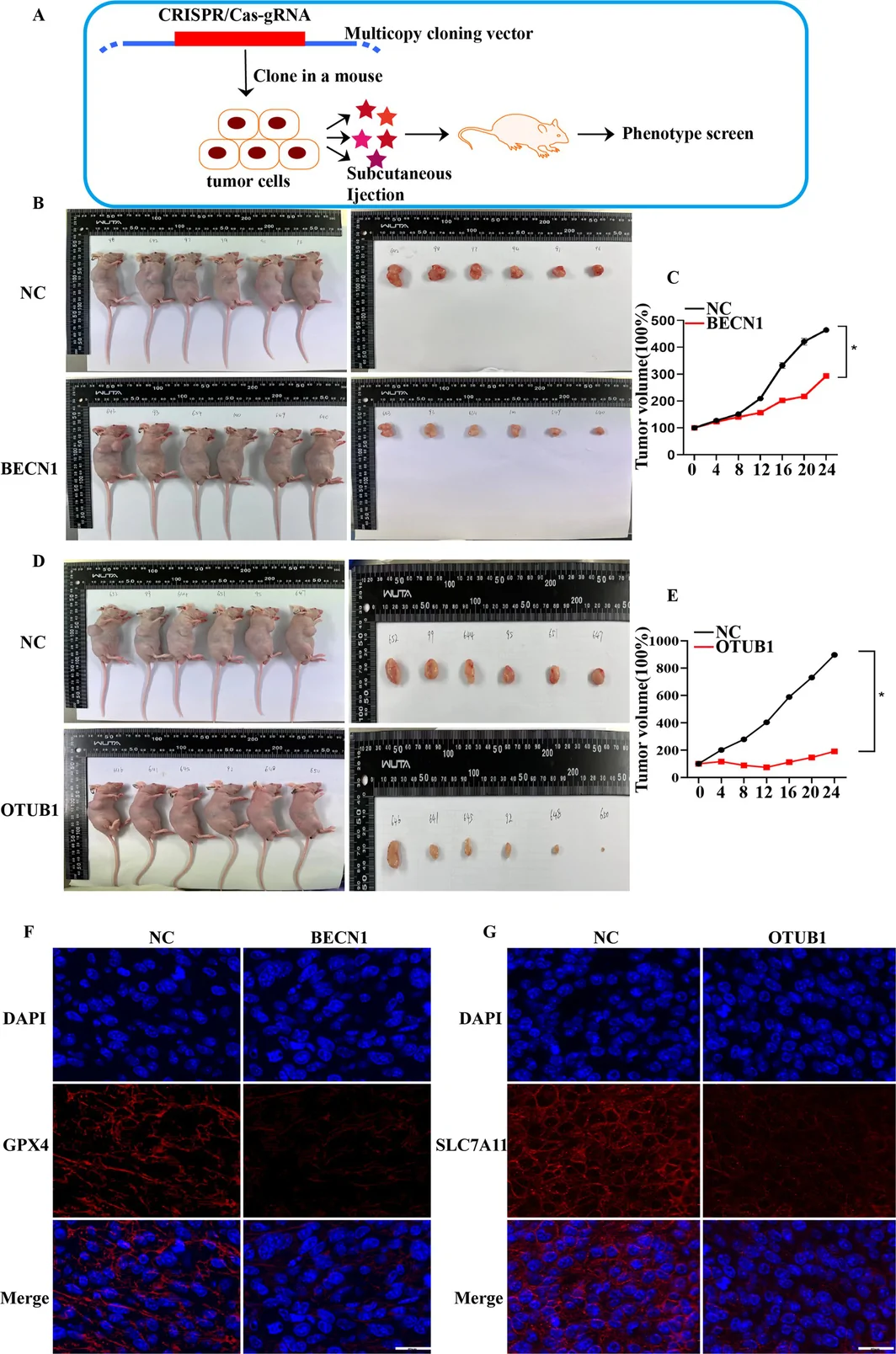
(A) Evaluation of the tools in vivo using BALB/c nude mice and B‐NDG mice. (B, D) Images of mice and dissected tumours in the control group compared to the group injection with transfected cells. (C, E) Change in tumour growth over time for the control group compared to the BECN1 upregulation and OTUB1 downregulation group respectively. (F, G) Representative immunofluorescence images and quantification of GPX4 and SLC7A11 in xenograft tumour tissues. GPX4 fluorescence was reduced in BECN1‐upregulated tumours compared with the VP64‐NC group, whereas SLC7A11 fluorescence was reduced in OTUB1‐downregulated tumours compared with the KRAB‐NC group. Data are presented as mean ± SD. *P < 0.05 versus the corresponding NC group.

Finally, we established an in vivo mouse model to elucidate the role of the BECN1 and OTUB1 ferroptosis pathways in BCa cell growth. In the xenograft tumour model, tumours derived from BECN1‐upregulated or OTUB1‐downregulated cells proliferated less than those in the control group, as measured by both tumour volume (Figure 7A–E) [25]. To further determine whether the in vivo antitumor effects were associated with ferroptosis‐related molecular changes, we performed immunofluorescence staining of GPX4 and SLC7A11 in xenograft tumour tissues. Compared with the VP64‐NC group, tumours with BECN1 upregulation showed markedly reduced GPX4 fluorescence signals. In parallel, compared with the KRAB‐NC group, tumours with OTUB1 downregulation showed reduced SLC7A11 fluorescence signals (Figure 7F,G). Since GPX4 and SLC7A11 are key components of the antioxidant defence system that protects cells from lipid peroxidation, these tumour‐level findings indicate that BECN1 upregulation and OTUB1 downregulation suppress the GPX4/SLC7A11 ferroptosis‐protective axis in vivo. Together with the in vitro Fer‐1/DFO rescue and C11‐BODIPY lipid ROS results, these data support that BECN1/OTUB1 regulation inhibits bladder cancer growth at least partly through a ferroptosis‐associated mechanism.

## 4 Discussion

BCa is the most common cancer of the urinary tract. The current treatments for BCa are often poor, and new techniques are urgently needed to target BCa in a more refined manner. Ferroptosis is a form of cell death that differs from apoptosis, and there is a growing body of research on the effect of targeting cellular lipid peroxidation. Some cancerous cells have been found to be particularly dependent on ferroptosis as a defence system to survive under conditions of high metabolic activity and the corresponding oxidative stress [26]. These data suggest that, under certain circumstances, ferroptosis represents a cancer vulnerability that can be exploited. At the same time, ferroptosis is also considered to be a key cell death response triggered by multiple mainstream cancer therapies, including radiotherapy, immunotherapy, chemotherapy, and targeted therapies [27], and thus targeting therapies against ferroptosis has great potential in cancer treatment research. In this study, we targeted ferroptosis‐related genes using CRISPR‐dCas9 under the control of the hTERT promoter to drive construct expression in cancerous cells. This system promoted ferroptosis‐associated molecular and phenotypic changes in BCa cells while showing limited effects on normal bladder cells, thereby exerting suppressive effects on bladder cancer cells.

First, we verified the correlation between the expression levels of the ferroptosis‐related protein, BECN1 and OTUB1 and tissue type, demonstrating their potential as an anti‐cancer target gene. BECN1 has been shown to interact with different molecular chaperones in various signalling pathways of cellular metabolism [28], such as in Liu et al. [29] where BECN1 was shown to be related to the GPX4 gene. OTUB1 was shown to regulate cancer‐related signalling pathways, and alterations in the expression of the OTUB1 gene were also able to affect the stability of SLC7A11, an important transporter on the ferroptosis pathway [30]. This finding suggests that targeting BECN1 and OTUB1 may affect ferroptosis‐related genes in UMUC‐3 and T24 cell lines, and thus may be a potential genetic target for BCa treatment.

To strengthen the evidence that BECN1 and OTUB1 regulation induced ferroptosis‐associated cell death, we performed pharmacological rescue experiments and lipid ROS assays. The reduction in cell viability caused by BECN1 upregulation or OTUB1 downregulation was partially rescued by Ferrostatin‐1 (Fer‐1) and deferoxamine (DFO), suggesting that the observed phenotype was at least partly dependent on lipid peroxidation and iron availability rather than nonspecific growth inhibition alone. Consistently, C11‐BODIPY staining showed increased lipid ROS accumulation in T24 cells following BECN1/OTUB1 regulation, and this increase was attenuated by Fer‐1 and DFO treatment. These findings support a ferroptosis‐associated mechanism downstream of the BECN1/GPX4 and OTUB1/SLC7A11 regulatory axes. In addition, because Annexin V/PI staining can reflect multiple forms of cell death and is not specific for apoptosis, we interpreted these data as Annexin V/PI‐positive cell death rather than definitive apoptotic death. The specificity of hTERT activity in cancer cells has been documented previously [19]. In this study, we confirmed that hTERT is specifically active in BCa cells. We designed a novel system using hTERT as a promoter for a CRISPR‐dCas9 construct that drives overexpression or suppression of the target genes, and applied it for the first time in the BCa cell lines UMUC‐3 and T24. Using this tool, we revealed the regulatory effects of BECN1 and OTUB1 on ferroptosis, with the regulation of these two genes significantly affecting the expression of genes involved in the ferroptosis pathway and promoting lipid peroxidation in cells. In vivo and in vitro experiments demonstrated the inhibitory effects of this regulatory system on the cells' abilities to proliferate, migrate, and invade. The in vivo xenograft findings further supported the proposed BECN1‐GPX4 and OTUB1‐SLC7A11 regulatory axes. Immunofluorescence staining of tumour tissues showed reduced GPX4 expression in BECN1‐upregulated tumours and reduced SLC7A11 expression in OTUB1‐downregulated tumours, consistent with the molecular changes observed in vitro. Since GPX4 and SLC7A11 are key components of the ferroptosis‐protective antioxidant system, their reduction suggests that BECN1 upregulation and OTUB1 downregulation weaken the antioxidant defence against lipid peroxidation in vivo. These results strengthen the mechanistic interpretation of the xenograft data and indicate that the observed tumour growth inhibition is accompanied by suppression of the GPX4/SLC7A11 ferroptosis‐protective axis rather than being inferred solely from tumour volume changes.

The quantitative model developed in this study aligns well with experimental observations, offering a robust framework for understanding the mechanistic roles of BECN1 and OTUB1 in ferroptosis. By quantifying the relationship between gene regulation and ferroptosis induction, the model provides a predictive tool for optimizing therapeutic strategies targeting these pathways. Moreover, our study demonstrated that the hTERT promoter can selectively target gene expression in cancerous cells without affecting normal tissues. Specifically, the hTERT‐promoter‐dCas9‐VP64 system successfully upregulated BECN1, while the hTERT‐promoter‐dCas9‐KRAB system downregulated OTUB1, leading to alterations in the expression of downstream genes GPX4 and SLC7A11. These changes triggered lipid peroxidation and subsequent cancer cell death. This highlights the potential of the hTERT‐based promoter combined with the CRISPR‐dCas9 system to modulate ferroptosis‐related proteins and induce lipid peroxidation in bladder cancer cells, paving the way for new targeted therapies in bladder cancer treatment.

Despite these promising results, the study has limitations. The long‐term effects and safety profile of the CRISPR‐dCas9 system, particularly regarding its immune response, warrant further investigation. Additionally, while our animal model results are encouraging, clinical studies are necessary to confirm the therapeutic efficacy and safety in human patients. Furthermore, this study did not specifically assess the risk of secondary tumours associated with hTERT use. Future research should thoroughly examine the appropriate hTERT dosage and its long‐term effects to ensure safety and minimise potential side effects.

## 5 Conclusion

In conclusion, our study establishes a novel and precise method for modulating ferroptosis in BCa cells using a CRISPR‐dCas9 system controlled by an hTERT promoter. This approach offers a promising strategy for developing targeted therapies, potentially improving the prognosis for BCa patients. Future research should focus on refining the delivery mechanisms and assessing the clinical applicability of this system to further advance bladder cancer treatment.

## Author Contributions

**Chaojie Xu:** software, investigation. **Chen Li:** formal analysis. **Ying Dong:** investigation, methodology, software, data curation, writing – original draft, writing – review and editing. **Shuanzhu Mou:** software. **Yuchen Liu:** supervision, funding acquisition, writing – review and editing, methodology. **Bing Yan:** investigation.

## Funding

This study was funded by National Key R&D Program of China (2021YFA0911600) and Shenzhen Science and Technology Program (Grant Nos. RCJC20221008092723011 and JCYJ20220818102001002).

## Ethics Statement

All animal experiments were conducted in accordance with the guidelines for the care and use of laboratory animals issued by the Ministry of Science and Technology of China, and approved by the Institutional Animal Care and Use Committee (IACUC) of Shenzhen topbiotech Institute (TOP‐IACUC‐2022‐0193).

## Consent

The authors have nothing to report.

## Conflicts of Interest

The authors declare no conflicts of interest.

## Supporting information


**Figure S1:** Fer‐1 reduces Annexin V‐positive cell death in BECN1‐upregulated T24 cells.
**Table S1:** Construction of virus vector hTERT‐promoter‐dCas9‐VP64 and hTERT‐promoter‐dCas9‐KRAB.
**Table S2:** Primer sequence.
**Table S3:** Maps of plasmids used in this study.

## Data Availability

The data that support the findings of this study are available from the corresponding author upon reasonable request.
