# Supplementary material for: Quantitative and Targeted Regulation of Ferroptosis in Bladder Cancer: Preclinical Study
Source: J Cell Mol Med. 2026 Aug 26;30(16):e71333. doi: 10.1111/jcmm.71333 (PMC13508133; doi:10.1111/jcmm.71333)
Supplement: Supplementary file 1 [file JCMM-30-e71333-s001.docx]

Supplementary Table:

**Supplementary Table 1: Construction of virus vector hTERT-promoter-dCas9-VP64 and hTERT-promoter-dCas9-KRAB**

| hTERT-promoter-dCas9-VP64: | AAACAAATTACAAAAATTCAAAATTTTCGGGTTTATTACAGGGACAGCAGAGATCCAGTTTGGTTAATTAATAAGTATTTACAAGACGAGGCTAACCTCCAGCGAGCGTGACAGCCCAGGGAGGGTGCGAGGCCTGTTCAAATGCTAGCTCCATAAATAAAGCAATTTCCTCCGGCAGTTTCTGAAAGTAGGAAAGGTTACATTTAAGGTTGCGTTTGTTAGCATTTCAGTGTTTGCCGACCTCAGCTACAGCATCCCTGCAAGGCCTCGGGAGACCCAGAAGTTTCTCGCCCCTTAGATCCAAACTTGAGCAACCCGGAGTCTGGATTCCTGGGAAGTCCTCAGCTGTCCTGCGGTTGTGCCGGGGCCCCAGGTCTGGAGGGGACCAGTGGCCGTGTGGCTTCTACTGCTGGGCTGGAAGTCGGGCCTCCTAGCTCTGCAGTCCGAGGCTTGGAGCCAGGTGCCTGGACCCCGAGGTTGCCCTCCACCCTGTGCGGGCGGGATGTGACCAGATGTTGGCCTCATCTGCCAGACAGAGTGCCGGGGCCCAGGGTCAAGGCCGTTGTGGCTGGTGTGAGGCGCCCGGTGCGCGGCCAGCAGGAGCGCCTGGCTCCATTTCCCACCCTTTCTCGACGGGACCGCCCCGGTGGGTGATTAACAGATTTGGGGTGGTTTGCTCATGGTGGGGACCCCTCGCCGCCTGAGAACCTGCAAAGAGAAATGACGGGCCTGTGTCAAGGAGCCCAAGTCGCGGGGAAGTGTTGCAGGGAGGCACTCCGGGAGGTCCCGCGTGCCCGTCCAGGGAGCAATGCGTCCTCGGGTTCGTCCCCAGCCGCGTCTACGCGCCTCCGTCCTCCCCTTCACGTCCGGCATTCGTGGTGCCCGGAGCCCGACGCCCCGCGTCCGGACCTGGAGGCAGCCCTGGGTCTCCGGATCAGGCCAGCGGCCAAAGGGTCGCCGCACGCACCTGTTCCCAGGGCCTCCACATCATGGCCCCTCCCTCGGGTTACCCCACAGCCTAGGCCGATTCGACCTCTCTCCGCTGGGGCCCTCGCTGGCGTCCCTGCACCCTGGGAGCGCGAGCGGCGCGCGGGCGGGGAAGCGCGGCCCAGACCCCCGGGTCCGCCCGGAGCAGCTGCGCTGTCGGGGCCAGGCCGGGCTCCCAGTGGATTCGCGGGCACAGACGCCCAGGACCGCGCTTCCCACGTGGCGGAGGGACTGGGGACCCGGGCACCCGTCCTGCCCCTTCACCTTCCAGCTCCGCCTCCTCCGCGCGGACCCCGCCCCGTCCCGACCCCTCCCGGGTCCCCGGCCCAGCCCCCTCCGGGCCCTCCCAGCCCCTCCCCTTCCTTTCCGCGGCCCCGCCCTCTCCTCGCGGCGCGAGTTTCAGGCAGCGCTGCGTCCTGCTGCGCACGTGGGAAGCCCTGGCCCCGGCCACCCCCGCGCGTACGGCCACCATGAAAAGGCCGGCGGCCACGAAAAAGGCCGGCCAGGCAAAAAAGAAAAAGGACAAGAAGTACAGCATCGGCCTGGCCATCGGCACCAACTCTGTGGGCTGGGCCGTGATCACCGACGAGTACAAGGTGCCCAGCAAGAAATTCAAGGTGCTGGGCAACACCGACCGGCACAGCATCAAGAAGAACCTGATCGGAGCCCTGCTGTTCGACAGCGGCGAAACAGCCGAGGCCACCCGGCTGAAGAGAACCGCCAGAAGAAGATACACCAGACGGAAGAACCGGATCTGCTATCTGCAAGAGATCTTCAGCAACGAGATGGCCAAGGTGGACGACAGCTTCTTCCACAGACTGGAAGAGTCCTTCCTGGTGGAAGAGGATAAGAAGCACGAGCGGCACCCCATCTTCGGCAACATCGTGGACGAGGTGGCCTACCACGAGAAGTACCCCACCATCTACCACCTGAGAAAGAAACTGGTGGACAGCACCGACAAGGCCGACCTGCGGCTGATCTATCTGGCCCTGGCCCACATGATCAAGTTCCGGGGCCACTTCCTGATCGAGGGCGACCTGAACCCCGACAACAGCGACGTGGACAAGCTGTTCATCCAGCTGGTGCAGACCTACAACCAGCTGTTCGAGGAAAACCCCATCAACGCCAGCGGCGTGGACGCCAAGGCCATCCTGTCTGCCAGACTGAGCAAGAGCAGACGGCTGGAAAATCTGATCGCCCAGCTGCCCGGCGAGAAGAAGAATGGCCTGTTCGGAAACCTGATTGCCCTGAGCCTGGGCCTGACCCCCAACTTCAAGAGCAACTTCGACCTGGCCGAGGATGCCAAACTGCAGCTGAGCAAGGACACCTACGACGACGACCTGGACAACCTGCTGGCCCAGATCGGCGACCAGTACGCCGACCTGTTTCTGGCCGCCAAGAACCTGTCCGACGCCATCCTGCTGAGCGACATCCTGAGAGTGAACACCGAGATCACCAAGGCCCCCCTGAGCGCCTCTATGATCAAGAGATACGACGAGCACCACCAGGACCTGACCCTGCTGAAAGCTCTCGTGCGGCAGCAGCTGCCTGAGAAGTACAAAGAGATTTTCTTCGACCAGAGCAAGAACGGCTACGCCGGCTACATTGACGGCGGAGCCAGCCAGGAAGAGTTCTACAAGTTCATCAAGCCCATCCTGGAAAAGATGGACGGCACCGAGGAACTGCTCGTGAAGCTGAACAGAGAGGACCTGCTGCGGAAGCAGCGGACCTTCGACAACGGCAGCATCCCCCACCAGATCCACCTGGGAGAGCTGCACGCCATTCTGCGGCGGCAGGAAGATTTTTACCCATTCCTGAAGGACAACCGGGAAAAGATCGAGAAGATCCTGACCTTCCGCATCCCCTACTACGTGGGCCCTCTGGCCAGGGGAAACAGCAGATTCGCCTGGATGACCAGAAAGAGCGAGGAAACCATCACCCCCTGGAACTTCGAGGAAGTGGTGGACAAGGGCGCTTCCGCCCAGAGCTTCATCGAGCGGATGACCAACTTCGATAAGAACCTGCCCAACGAGAAGGTGCTGCCCAAGCACAGCCTGCTGTACGAGTACTTCACCGTGTATAACGAGCTGACCAAAGTGAAATACGTGACCGAGGGAATGAGAAAGCCCGCCTTCCTGAGCGGCGAGCAGAAAAAGGCCATCGTGGACCTGCTGTTCAAGACCAACCGGAAAGTGACCGTGAAGCAGCTGAAAGAGGACTACTTCAAGAAAATCGAGTGCTTCGACTCCGTGGAAATCTCCGGCGTGGAAGATCGGTTCAACGCCTCCCTGGGCACATACCACGATCTGCTGAAAATTATCAAGGACAAGGACTTCCTGGACAATGAGGAAAACGAGGACATTCTGGAAGATATCGTGCTGACCCTGACACTGTTTGAGGACAGAGAGATGATCGAGGAACGGCTGAAAACCTATGCCCACCTGTTCGACGACAAAGTGATGAAGCAGCTGAAGCGGCGGAGATACACCGGCTGGGGCAGGCTGAGCCGGAAGCTGATCAACGGCATCCGGGACAAGCAGTCCGGCAAGACAATCCTGGATTTCCTGAAGTCCGACGGCTTCGCCAACAGAAACTTCATGCAGCTGATCCACGACGACAGCCTGACCTTTAAAGAGGACATCCAGAAAGCCCAGGTGTCCGGCCAGGGCGATAGCCTGCACGAGCACATTGCCAATCTGGCCGGCAGCCCCGCCATTAAGAAGGGCATCCTGCAGACAGTGAAGGTGGTGGACGAGCTCGTGAAAGTGATGGGCCGGCACAAGCCCGAGAACATCGTGATCGAaATGGCCAGAGAGAACCAGACCACCCAGAAGGGACAGAAGAACAGCCGCGAGAGAATGAAGCGGATCGAAGAGGGCATCAAAGAGCTGGGCAGCCAGATCCTGAAAGAACACCCCGTGGAAAACACCCAGCTGCAGAACGAGAAGCTGTACCTGTACTACCTGCAGAATGGGCGGGATATGTACGTGGACCAGGAACTGGACATCAACCGGCTGTCCGACTACGATGTGGACCATATCGTGCCTCAGAGCTTTCTGAAGGACGACTCCATCGACAACAAGGTGCTGACCAGAAGCGACAAGGCCCGGGGCAAGAGCGACAACGTGCCCTCCGAAGAGGTCGTGAAGAAGATGAAGAACTACTGGCGGCAGCTGCTGAACGCCAAGCTGATTACCCAGAGAAAGTTCGACAATCTGACCAAGGCCGAGAGAGGCGGCCTGAGCGAACTGGATAAGGCCGGCTTCATCAAGAGACAGCTGGTGGAAACCCGGCAGATCACAAAGCACGTGGCACAGATCCTGGACTCCCGGATGAACACTAAGTACGACGAGAATGACAAGCTGATCCGGGAAGTGAAAGTGATCACCCTGAAGTCCAAGCTGGTGTCCGATTTCCGGAAGGATTTCCAGTTTTACAAAGTGCGCGAGATCAACAACTACCACCACGCCCACGACGCCTACCTGAACGCCGTCGTGGGAACCGCCCTGATCAAAAAGTACCCTAAGCTGGAAAGCGAGTTCGTGTACGGCGACTACAAGGTGTACGACGTGCGGAAGATGATCGCCAAGAGCGAGCAGGAAATCGGCAAGGCTACCGCCAAGTACTTCTTCTACAGCAACATCATGAACTTTTTCAAGACCGAGATTACCCTGGCCAACGGCGAGATCCGGAAGCGGCCTCTGATCGAGACAAACGGCGAAACCGGGGAGATCGTGTGGGATAAGGGCCGGGATTTTGCCACCGTGCGGAAAGTGCTGAGCATGCCCCAAGTGAATATCGTGAAAAAGACCGAGGTGCAGACAGGCGGCTTCAGCAAAGAGTCTATCCTGCCCAAGAGGAACAGCGATAAGCTGATCGCCAGAAAGAAGGACTGGGACCCTAAGAAGTACGGCGGCTTCGACAGCCCCACCGTGGCCTATTCTGTGCTGGTGGTGGCCAAAGTGGAAAAGGGCAAGTCCAAGAAACTGAAGAGTGTGAAAGAGCTGCTGGGGATCACCATCATGGAAAGAAGCAGCTTCGAGAAGAATCCCATCGACTTTCTGGAAGCCAAGGGCTACAAAGAAGTGAAAAAGGACCTGATCATCAAGCTGCCTAAGTACTCCCTGTTCGAGCTGGAAAACGGCCGGAAGAGAATGCTGGCCTCTGCCGGCGAACTGCAGAAGGGAAACGAACTGGCCCTGCCCTCCAAATATGTGAACTTCCTGTACCTGGCCAGCCACTATGAGAAGCTGAAGGGCTCCCCCGAGGATAATGAGCAGAAACAGCTGTTTGTGGAACAGCACAAGCACTACCTGGACGAGATCATCGAGCAGATCAGCGAGTTCTCCAAGAGAGTGATCCTGGCCGACGCTAATCTGGACAAAGTGCTGTCCGCCTACAACAAGCACCGGGATAAGCCCATCAGAGAGCAGGCCGAGAATATCATCCACCTGTTTACCCTGACCAATCTGGGAGCCCCTGCCGCCTTCAAGTACTTTGACACCACCATCGACCGGAAGAGGTACACCAGCACCAAAGAGGTGCTGGACGCCACCCTGATCCACCAGAGCATCACCGGCCTGTACGAGACACGGATCGACCTGTCTCAGCTGGGAGGCGACCTCGAGAGCGCTGGAGGAGGTGGAAGCGGAGGAGGAGGAAGCGGAGGAGGAGGTAGCGGACCTAAGAAAAAGAGGAAGGTGGCGGCCGCTGGATCCGGACGGGCTGACGCATTGGACGATTTTGATCTGGATATGCTGGGAAGTGACGCCCTCGATGATTTTGACCTTGACATGCTTGGTTCGGATGCCCTTGATGACTTTGACCTCGACATGCTCGGCAGTGACGCCCTTGATGATTTCGACCTGGACATGCTGTAAGAATTCCTGTGGAATGTGTGTCAGTTAGGGTGTGGAAAGTCCCCAGGCTCCCCAGCAGGCAGAAGTATGCAAAGCATGCATCTCAATTAGTCAGCAACCAGGTGTGGAAAGTCCCCAGGCTCCCCAGCAGGCAGAAGTATGCAAAGCATGCATCTCAATTAGTCAGCAACCATAGTCCCGCCCCTAACTCCGCCCA |
| --- | --- |
| hTERT-promoter-dCas9-KRAB: | AAACAAATTACAAAAATTCAAAATTTTCGGGTTTATTACAGGGACAGCAGAGATCCAGTTTGGTTAATTAATAAGTATTTACAAGACGAGGCTAACCTCCAGCGAGCGTGACAGCCCAGGGAGGGTGCGAGGCCTGTTCAAATGCTAGCTCCATAAATAAAGCAATTTCCTCCGGCAGTTTCTGAAAGTAGGAAAGGTTACATTTAAGGTTGCGTTTGTTAGCATTTCAGTGTTTGCCGACCTCAGCTACAGCATCCCTGCAAGGCCTCGGGAGACCCAGAAGTTTCTCGCCCCTTAGATCCAAACTTGAGCAACCCGGAGTCTGGATTCCTGGGAAGTCCTCAGCTGTCCTGCGGTTGTGCCGGGGCCCCAGGTCTGGAGGGGACCAGTGGCCGTGTGGCTTCTACTGCTGGGCTGGAAGTCGGGCCTCCTAGCTCTGCAGTCCGAGGCTTGGAGCCAGGTGCCTGGACCCCGAGGTTGCCCTCCACCCTGTGCGGGCGGGATGTGACCAGATGTTGGCCTCATCTGCCAGACAGAGTGCCGGGGCCCAGGGTCAAGGCCGTTGTGGCTGGTGTGAGGCGCCCGGTGCGCGGCCAGCAGGAGCGCCTGGCTCCATTTCCCACCCTTTCTCGACGGGACCGCCCCGGTGGGTGATTAACAGATTTGGGGTGGTTTGCTCATGGTGGGGACCCCTCGCCGCCTGAGAACCTGCAAAGAGAAATGACGGGCCTGTGTCAAGGAGCCCAAGTCGCGGGGAAGTGTTGCAGGGAGGCACTCCGGGAGGTCCCGCGTGCCCGTCCAGGGAGCAATGCGTCCTCGGGTTCGTCCCCAGCCGCGTCTACGCGCCTCCGTCCTCCCCTTCACGTCCGGCATTCGTGGTGCCCGGAGCCCGACGCCCCGCGTCCGGACCTGGAGGCAGCCCTGGGTCTCCGGATCAGGCCAGCGGCCAAAGGGTCGCCGCACGCACCTGTTCCCAGGGCCTCCACATCATGGCCCCTCCCTCGGGTTACCCCACAGCCTAGGCCGATTCGACCTCTCTCCGCTGGGGCCCTCGCTGGCGTCCCTGCACCCTGGGAGCGCGAGCGGCGCGCGGGCGGGGAAGCGCGGCCCAGACCCCCGGGTCCGCCCGGAGCAGCTGCGCTGTCGGGGCCAGGCCGGGCTCCCAGTGGATTCGCGGGCACAGACGCCCAGGACCGCGCTTCCCACGTGGCGGAGGGACTGGGGACCCGGGCACCCGTCCTGCCCCTTCACCTTCCAGCTCCGCCTCCTCCGCGCGGACCCCGCCCCGTCCCGACCCCTCCCGGGTCCCCGGCCCAGCCCCCTCCGGGCCCTCCCAGCCCCTCCCCTTCCTTTCCGCGGCCCCGCCCTCTCCTCGCGGCGCGAGTTTCAGGCAGCGCTGCGTCCTGCTGCGCACGTGGGAAGCCCTGGCCCCGGCCACCCCCGCGCGTACGGCCACCATGAAAAGGCCGGCGGCCACGAAAAAGGCCGGCCAGGCAAAAAAGAAAAAGGACAAGAAGTACAGCATCGGCCTGGCCATCGGCACCAACTCTGTGGGCTGGGCCGTGATCACCGACGAGTACAAGGTGCCCAGCAAGAAATTCAAGGTGCTGGGCAACACCGACCGGCACAGCATCAAGAAGAACCTGATCGGAGCCCTGCTGTTCGACAGCGGCGAAACAGCCGAGGCCACCCGGCTGAAGAGAACCGCCAGAAGAAGATACACCAGACGGAAGAACCGGATCTGCTATCTGCAAGAGATCTTCAGCAACGAGATGGCCAAGGTGGACGACAGCTTCTTCCACAGACTGGAAGAGTCCTTCCTGGTGGAAGAGGATAAGAAGCACGAGCGGCACCCCATCTTCGGCAACATCGTGGACGAGGTGGCCTACCACGAGAAGTACCCCACCATCTACCACCTGAGAAAGAAACTGGTGGACAGCACCGACAAGGCCGACCTGCGGCTGATCTATCTGGCCCTGGCCCACATGATCAAGTTCCGGGGCCACTTCCTGATCGAGGGCGACCTGAACCCCGACAACAGCGACGTGGACAAGCTGTTCATCCAGCTGGTGCAGACCTACAACCAGCTGTTCGAGGAAAACCCCATCAACGCCAGCGGCGTGGACGCCAAGGCCATCCTGTCTGCCAGACTGAGCAAGAGCAGACGGCTGGAAAATCTGATCGCCCAGCTGCCCGGCGAGAAGAAGAATGGCCTGTTCGGCAACCTGATTGCCCTGAGCCTGGGCCTGACCCCCAACTTCAAGAGCAACTTCGACCTGGCCGAGGATGCCAAACTGCAGCTGAGCAAGGACACCTACGACGACGACCTGGACAACCTGCTGGCCCAGATCGGCGACCAGTACGCCGACCTGTTTCTGGCCGCCAAGAACCTGTCCGACGCCATCCTGCTGAGCGACATCCTGAGAGTGAACACCGAGATCACCAAGGCCCCCCTGAGCGCCTCTATGATCAAGAGATACGACGAGCACCACCAGGACCTGACCCTGCTGAAAGCTCTCGTGCGGCAGCAGCTGCCTGAGAAGTACAAAGAGATTTTCTTCGACCAGAGCAAGAACGGCTACGCCGGCTACATTGACGGCGGAGCCAGCCAGGAAGAGTTCTACAAGTTCATCAAGCCCATCCTGGAAAAGATGGACGGCACCGAGGAACTGCTCGTGAAGCTGAACAGAGAGGACCTGCTGCGGAAGCAGCGGACCTTCGACAACGGCAGCATCCCCCACCAGATCCACCTGGGAGAGCTGCACGCCATTCTGCGGCGGCAGGAAGATTTTTACCCATTCCTGAAGGACAACCGGGAAAAGATCGAGAAGATCCTGACCTTCCGCATCCCCTACTACGTGGGCCCTCTGGCCAGGGGAAACAGCAGATTCGCCTGGATGACCAGAAAGAGCGAGGAAACCATCACCCCCTGGAACTTCGAGGAAGTGGTGGACAAGGGCGCTTCCGCCCAGAGCTTCATCGAGCGGATGACCAACTTCGATAAGAACCTGCCCAACGAGAAGGTGCTGCCCAAGCACAGCCTGCTGTACGAGTACTTCACCGTGTATAACGAGCTGACCAAAGTGAAATACGTGACCGAGGGAATGAGAAAGCCCGCCTTCCTGAGCGGCGAGCAGAAAAAGGCCATCGTGGACCTGCTGTTCAAGACCAACCGGAAAGTGACCGTGAAGCAGCTGAAAGAGGACTACTTCAAGAAAATCGAGTGCTTCGACTCCGTGGAAATCTCCGGCGTGGAAGATCGGTTCAACGCCTCCCTGGGCACATACCACGATCTGCTGAAAATTATCAAGGACAAGGACTTCCTGGACAATGAGGAAAACGAGGACATTCTGGAAGATATCGTGCTGACCCTGACACTGTTTGAGGACAGAGAGATGATCGAGGAACGGCTGAAAACCTATGCCCACCTGTTCGACGACAAAGTGATGAAGCAGCTGAAGCGGCGGAGATACACCGGCTGGGGCAGGCTGAGCCGGAAGCTGATCAACGGCATCCGGGACAAGCAGTCCGGCAAGACAATCCTGGATTTCCTGAAGTCCGACGGCTTCGCCAACAGAAACTTCATGCAGCTGATCCACGACGACAGCCTGACCTTTAAAGAGGACATCCAGAAAGCCCAGGTGTCCGGCCAGGGCGATAGCCTGCACGAGCACATTGCCAATCTGGCCGGCAGCCCCGCCATTAAGAAGGGCATCCTGCAGACAGTGAAGGTGGTGGACGAGCTCGTGAAAGTGATGGGCCGGCACAAGCCCGAGAACATCGTGATCGAAATGGCCAGAGAGAACCAGACCACCCAGAAGGGACAGAAGAACAGCCGCGAGAGAATGAAGCGGATCGAAGAGGGCATCAAAGAGCTGGGCAGCCAGATCCTGAAAGAACACCCCGTGGAAAACACCCAGCTGCAGAACGAGAAGCTGTACCTGTACTACCTGCAGAATGGGCGGGATATGTACGTGGACCAGGAACTGGACATCAACCGGCTGTCCGACTACGATGTGGACCACATCGTGCCTCAGAGCTTTCTGAAGGACGACTCCATCGACAACAAGGTGCTGACCAGAAGCGACAAGGCCCGGGGCAAGAGCGACAACGTGCCCTCCGAAGAGGTCGTGAAGAAGATGAAGAACTACTGGCGGCAGCTGCTGAACGCCAAGCTGATTACCCAGAGAAAGTTCGACAATCTGACCAAGGCCGAGAGAGGCGGCCTGAGCGAACTGGATAAGGCCGGCTTCATCAAGAGACAGCTGGTGGAAACCCGGCAGATCACAAAGCACGTGGCACAGATCCTGGACTCCCGGATGAACACTAAGTACGACGAGAATGACAAGCTGATCCGGGAAGTGAAAGTGATCACCCTGAAGTCCAAGCTGGTGTCCGATTTCCGGAAGGATTTCCAGTTTTACAAAGTGCGCGAGATCAACAACTACCACCACGCCCACGACGCCTACCTGAACGCCGTCGTGGGAACCGCCCTGATCAAAAAGTACCCTAAGCTGGAAAGCGAGTTCGTGTACGGCGACTACAAGGTGTACGACGTGCGGAAGATGATCGCCAAGAGCGAGCAGGAAATCGGCAAGGCTACCGCCAAGTACTTCTTCTACAGCAACATCATGAACTTTTTCAAGACCGAGATTACCCTGGCCAACGGCGAGATCCGGAAGCGGCCTCTGATCGAGACAAACGGCGAAACCGGGGAGATCGTGTGGGATAAGGGCCGGGATTTTGCCACCGTGCGGAAAGTGCTGAGCATGCCCCAAGTGAATATCGTGAAAAAGACCGAGGTGCAGACAGGCGGCTTCAGCAAAGAGTCTATCCTGCCCAAGAGGAACAGCGATAAGCTGATCGCCAGAAAGAAGGACTGGGACCCTAAGAAGTACGGCGGCTTCGACAGCCCCACCGTGGCCTATTCTGTGCTGGTGGTGGCCAAAGTGGAAAAGGGCAAGTCCAAGAAACTGAAGAGTGTGAAAGAGCTGCTGGGGATCACCATCATGGAAAGAAGCAGCTTCGAGAAGAATCCCATCGACTTTCTGGAAGCCAAGGGCTACAAAGAAGTGAAAAAGGACCTGATCATCAAGCTGCCTAAGTACTCCCTGTTCGAGCTGGAAAACGGCCGGAAGAGAATGCTGGCCTCTGCCGGCGAACTGCAGAAGGGAAACGAACTGGCCCTGCCCTCCAAATATGTGAACTTCCTGTACCTGGCCAGCCACTATGAGAAGCTGAAGGGCTCCCCCGAGGATAATGAGCAGAAACAGCTGTTTGTGGAACAGCACAAGCACTACCTGGACGAGATCATCGAGCAGATCAGCGAGTTCTCCAAGAGAGTGATCCTGGCCGACGCTAATCTGGACAAAGTGCTGTCCGCCTACAACAAGCACCGGGATAAGCCCATCAGAGAGCAGGCCGAGAATATCATCCACCTGTTTACCCTGACCAATCTGGGAGCCCCTGCCGCCTTCAAGTACTTTGACACCACCATCGACCGGAAGAGGTACACCAGCACCAAAGAGGTGCTGGACGCCACCCTGATCCACCAGAGCATCACCGGCCTGTACGAGACACGGATCGACCTGTCTCAGCTGGGAGGCGACAGCGCTGGAGGAGGTGGAAGCGGAGGAGGAGGAAGCGGAGGAGGAGGTAGCGGACCTAAGAAAAAGAGGAAGGTGGCGGCCGCTGGATCCATGGATGCTAAGTCACTAACTGCCTGGTCCCGGACACTGGTGACCTTCAAGGATGTATTTGTGGACTTCACCAGGGAGGAGTGGAAGCTGCTGGACACTGCTCAGCAGATCGTGTACAGAAATGTGATGCTGGAGAACTATAAGAACCTGGTTTCCTTGGGTTATCAGCTTACTAAGCCAGATGTGATCCTCCGGTTGGAGAAGGGAGAAGAGCCCTAAGAATTCCTGTGGAATGTGTGTCAGTTAGGGTGTGGAAAGTCCCCAGGCTCCCCAGCAGGCAGAAGTATGCAAAGCATGCATCTCAATTAGTCAGCAACCAGGTGTGGAAAGTCCCCAGGCTCCCCAGCAGGCAGAAGTATGCAAAGCATGCATCTCAATTAGTCAGCAACCATAGTCCCGCCCCTAACTCCGCCCATCCCGCCCCTAACTCCGCCCAGTTCCGCCCATTCTCCGCCCCATGGCTGACTAATTTTTTTTATTTATGCAGAGGCCGAGGCCGCCTCTGCCTCTGAGCTATTCCAGAAGTAGTGAGGAGGCTTTTTTGGAGGCCTAGGCTTTTGCAAAAAGCTC |

**Supplementary Table 2:** **Primer sequence.**

| **sgRNA of BECN1** | CTCCGCTATTCTCTAAATTC |
| --- | --- |
| **sgRNA of OTUB1** | CACCGGGAGGACGGCAATTGGGCAAC |

**Supplementary Table 3：Maps of plasmids used in this study.**

| **Item** | Vector map / Electrophoresis result |
| --- | --- |
| **Lenti-CAS9-puro** |  |
| hTERT-promoter-dCas9-VP64 |  |
| Target lentiviral vector |  |
| Enzyme digestion result of Carrier |   1#: 10kb Marker (Bands from top to bottom: 10kb, 8kb, 6kb, 5kb, 4kb, 3.5kb, 3kb, 2.5kb, 2kb, 1.5kb, 1kb, 750bp, 500bp, 250bp)  2#: vector digestion product  3#: Unselected vector |
| PCR result of Target gene fragment |   Marker：Bands from top to bottom 10kb、8kb、6kb、5kb、4kb、3.5kb、3kb、2.5kb、2kb、1.5kb、1kb、750bp、500bp、250bp |
| PCR result of recombinant plasmids |   1#: Negative control (ddH2O)  2#: negative control (no-load self-linked control group)  3#: Positive control (GAPDH)  4#: Marker in the order of 5 kb, 3 kb, 2 kb, 1.5 kb, 1 Kb, 750 bp, 500 bp, 250 bp, 100 bp from top to bottom  5-12#:Transformants 1-8 |

**Supplementary Figure S1：Fer-1 reduces Annexin V-positive cell death in BECN1-upregulated T24 cells.**

Supplementary Figure S1. Fer-1 reduces Annexin V-positive cell death in BECN1-upregulated T24 cells. Representative Annexin V/PI flow cytometry plots and quantification showing that BECN1 upregulation increased Annexin V-positive cell death in T24 cells, whereas Fer-1 treatment partially reduced this population. The percentage of Annexin V-positive cells was calculated as Q2 + Q3. Data are presented as mean ± SD. ns, not significant; *P<0.05; **P<0.01; ***P<0.001.
